# The role of HFE genotype in macrophage phenotype

**DOI:** 10.1186/s12974-018-1057-0

**Published:** 2018-02-01

**Authors:** Anne M. Nixon, Elizabeth Neely, Ian A. Simpson, James R. Connor

**Affiliations:** 1 Department of Neurosurgery, The Pennsylvania State University College of Medicine, M.S. Hershey Medical Center, Hershey, PA 17033 USA; 2 Department of Neural and Behavior Science, The Pennsylvania State University College of Medicine, M.S. Hershey Medical Center, Hershey, 17033 PA USA

**Keywords:** Iron, Macrophages, Microglia, HFE, H63D, H67D, Inflammation

## Abstract

**Background:**

Iron regulation is essential for cellular energy production. Loss of cellular iron homeostasis has critical implications for both normal function and disease progression. The H63D variant of the HFE gene is the most common gene variant in Caucasians. The resulting mutant protein alters cellular iron homeostasis and is associated with a number of neurological diseases and cancer. In the brain, microglial and infiltrating macrophages are critical to maintaining iron homeostasis and modulating inflammation associated with the pathogenic process in multiple diseases. This study addresses whether HFE genotype affects macrophage function and the implications of these findings for disease processes.

**Methods:**

Bone marrow macrophages were isolated from wildtype and H67D HFE knock-in mice. The H67D gene variant in mice is the human equivalent of the H63D variant. Upon differentiation, the macrophages were used to analyze iron regulatory proteins, cellular iron release, migration, phagocytosis, and cytokine expression.

**Results:**

The results of this study demonstrate that the H67D HFE genotype significantly impacts a number of critical macrophage functions. Specifically, fundamental activities such as proliferation in response to iron exposure, L-ferritin expression in response to iron loading, secretion of BMP6 and cytokines, and migration and phagocytic activity were all found to be impacted by genotype. Furthermore, we demonstrated that exposure to apo-Tf (iron-poor transferrin) can increase the release of iron from macrophages. In normal conditions, 70% of circulating transferrin is unsaturated. Therefore, the ability of apo-Tf to induce iron release could be a major regulatory mechanism for iron release from macrophages.

**Conclusions:**

These studies demonstrate that the HFE genotype impacts fundamental components of macrophage phenotype that could alter their role in degenerative and reparative processes in neurodegenerative disorders.

## Background

Iron is a critical cofactor in many biological and physiological processes [1]. Therefore, iron mismanagement can lead to dysfunction and damage to multiple systems. In recent years, particular interest has been developed in the HFE (high iron) gene. The HFE gene encodes the HFE protein, a transmembrane glycoprotein similar to the major histocompatibility complex (MHC) molecules [2]. HFE contributes to the regulation of iron through its ability to bind to transferrin receptors on cellular membranes [3, 4]. There are three known polymorphisms of the HFE gene: H63D, C282Y, and S56C. The C282Y variant is only present in < 2% of the population but is found in 90–95% of hemochromatosis patients [2, 5–8]. The H63D variant is the most common gene variant in Caucasians being reported in as high as 15–20% of the population [5, 8]. Our interest in the H63D HFE variant began with studies by our group and others reporting that this gene variant is increased in neurodegenerative diseases such as Alzheimer’s disease (AD) and amyotrophic lateral sclerosis (ALS) [9–11]. When mice with the H67D HFE gene variant (mouse homolog of H63D) are bred with the SOD1 mutant mouse model of ALS the result is accelerated disease progression [12]. These double transgenic mice have increased staining for microglia suggesting greater activation of microglia could contribute to the accelerated disease process. With normal aging, the H67D mice have an increase in ferritin expression in the brain which is associated with increased microglial staining profile [13, 14].

One of the key phenotypes associated with the HFE gene variant is that reticuloendothelial cells, specifically macrophages, are iron-poor [15, 16]. Macrophages normally play an important role in iron homeostasis through phagocytosis of debris and recycling iron [17–20]. Therefore, altered iron handling by macrophages and brain microglia could be part of the mechanism by which the H63D HFE variant may impact disease states. Iron status and microglial function have been demonstrated by a number of studies. The evidence is compelling that decreasing iron results in decreasing pro-inflammatory activity [21–23], whereas increasing iron results in increased pro-inflammation [24, 25] and decreased microglial phagocytic activity [24]. The HFE genotype status can be expected then to alter macrophage and microglia phenotype because the mutated HFE protein reportedly limits iron uptake in macrophages [26, 27]. In the brain, microglia are the resident macrophage and play a critical role in neurodegenerative diseases [28]. For example, both iron accumulation and increase in microglia and infiltrating macrophages occur in the brain with age which is thought to underlie the age-related increase in neuroinflammation and production of free radicals in the brain [29]. Our laboratory has been focused on the role of iron in neurodegenerative disease and normal brain function. In this study, we use macrophages as a model for interrogating the HFE genotype impact. Despite microglia and macrophage structural differences, they share important biological functions, such as iron loading with activation [30, 31], mediating inflammation, and recruitment of monocytes [32]. Microglia and infiltrating macrophages have a key role in neurodegenerative diseases, such as AD, multiple sclerosis, ALS, and spinal cord injury. For example, macrophages have been shown to be more efficient at clearing Aβ plaque than microglia [33], accumulate excess iron upon activation of TLR4 within iron overload sites of CNS injury [34], and migrate from the periphery to CNS where they may cause increased inflammation or relapse in MS patients [35].

Lastly, the HFE genotype is linked to increased frequency of cancer and macrophages are known to infiltrate brain tumors as important contributors to the tumor microenvironment [36–39]. Thus, HFE mediated iron regulation in macrophages and microglia, and its relationship to inflammatory responses may provide a mechanistic link between otherwise unrelated diseases.

## Methods

### Mouse colony

C57BL/6 J × 129 mice (12-month-old males) were utilized for this study. As previously described, the mice expressed either wildtype (WT) or the H67D HFE gene variant, the latter is a homolog for the human H63D HFE mutation [40]. The mice were maintained, in-house, in an animal facility at The Pennsylvania State University, College of Medicine. All procedures were approved by the Pennsylvania State University, College of Medicine, Institutional Animal Care and Use Committee, Protocol 04-166.

### Primary macrophage culture

Bone marrow-derived cells were extracted and cultured, as previously described [41]. Briefly, the mice were sacrificed by cervical dislocation and the femurs and tibias were removed, and epiphyses excised. The bone marrow was then flushed using Dulbecco’s phosphate-buffered saline (DPBS) (Corning; Manassas, VA). The resulting cell suspension was passed through a 40-μm cell strainer. The bone marrow cells were then plated in 100-mm^2^ tissue culture plates at a concentration of 8 × 10^6^ cells/plate. The cells were maintained in Dulbecco’s Modified Eagle’s Medium (DMEM) (Invitrogen; Grand Island, NY) with 10% fetal bovine serum (FBS) (Gemini Bio Products; West Sacramento, CA), 1% penicillin-streptomycin (Invitrogen; Grand Island, NY), and 10 ng/ml macrophage colony stimulating factor (M-CSF) (R&D Systems; Minneapolis, MN); and incubated at 37 °C under an atmosphere of 5% CO_2_ with humidified air. M-CSF was added to the cell culture media to allow differentiation of the bone marrow cells to bone marrow-derived macrophages (BMMs). Verification of bone marrow macrophage differentiation was confirmed through flow cytometry using macrophage surface and intracellular markers F4/80, CD11b, and CD68, as previously described [41]. Briefly, differentiated macrophages (1.2 × 10^7^ cells/sample) were resuspended in DPBS. Fc receptor blocking antibody (eBioscience; San Diego, CA) was added to each cell sample to prevent nonspecific antibody staining. Subsequently, anti-F4/80-FITC (BioLegend; San Diego, CA) and anti-CD11b-APC (BioLegend; San Diego, CA) were separately added to two of the cell suspensions and incubated at 4°C, in the dark for 30 min to prevent photobleaching. All cell samples were resuspended in BD Cytofix/Cytoperm reagent (BD Biosciences; San Jose, CA) and incubated for 20 min in the dark at 4 °C. The cell samples were then washed and resuspended with BD Perm/Wash buffer (BD Biosciences; San Jose, CA). Anti-CD68-PE (BioLegend; San Diego, CA) was then added to an unstained cell sample, and incubated for 30 min at 4 °C, in the dark for 30 min. Lastly, the cell samples were washed and subjected to flow cytometry using BD FACSCalibur (BD Biosciences; San Jose, CA). The resulting data were analyzed using FlowJo software in which fluorescence intensity of macrophage-specific markers were compared to the fluorescence intensity of unstained control cells.

### MTT and LDH assays

To determine the viability of the macrophages, 3-(4,5-dimethylthiazol-2-Yl)-2,5-diphenyltetrazolium Bromide (MTT) and lactate dehydrogenase (LDH) were used. Following differentiation, the macrophage cultures were washed with DPBS and divided into three different groups: (1) control media (same as culture media), (2) media supplemented with 300 μM ferric ammonia citrate (FAC) (Sigma Aldrich; St. Louis, MO) for iron loading, or (3) media supplemented with 300 μm deferoxamine (DFO) (Sigma Aldrich; St. Louis, MO), an iron chelator. Following a 24-h incubation period, the cells were washed with DPBS. The media in all groups was then replaced with control media and the cells incubated for an additional 24-h. Subsequently, cell viability was assessed using MTT (Roche; Basal, Switzerland) and LDH (Roche; Basal, Switzerland) assays, according to manufacturer’s instructions. Fluorescence was measured on a SpectraMax Gemini EM plate reader (Molecular Devices; Sunnyvale, CA).

### Enzyme linked Immunosorbent assays (ELISAs)

The macrophages were cultured and treated as described above. Following differentiation, the macrophages were lysed in RIPA buffer, (Sigma Aldrich; St. Louis, MO) that included a 1:100 dilution of protease inhibitors (Sigma Aldrich; St. Louis, MO). Protein concentrations were determined using a BCA protein assay kit (Pierce; Rockford, IL).

#### Iron proteins

The cell lysates were used to measure the amount of iron proteins in wildtype and H67D macrophages. We performed enzyme-linked immunosorbent assays (ELISAs) for the iron proteins: L-ferritin (Abcam; Cambridge, UK), H-ferritin (Mybiosource; San Diego, CA), transferrin receptor (BlueGene; Shanghai, China), and ferroportin (Cloud-Clone Corp; Houston, TX), according to their manufacturer’s instructions. Absorbance of the cell lysates was measured using a SpectraMax 340PC plate reader (Molecular Devices; Sunnyvale, CA).

#### Bone morphogenetic protein 6 (BMP6) and bone morphogenetic type I receptor (ALK3)

To quantify the amount of BMP6 and ALK3 within the macrophages, the cells were harvested and lysed as described previously. In addition, the amount of BMP6 secreted from the macrophages was determined from the media collected at the time of cell harvest. The samples were analyzed with a BMP6 (DL Develop; Wuxi, Jiangsu, China) or ALK3 (Mybiosource; San Diego, CA) specific ELISA according to manufacturer’s instructions. The absorbance of the cell lysates and cell culture media was measured on a SpectraMax 340PC plate reader, (Molecular Devices; Sunnyvale, CA).

### Phagocytosis assay

For the phagocytosis assay, 1 × 10^4^ macrophages/wells were plated on a black bottom 96-well plate. The cells were incubated with cell culture media for 24-h and then the media was replaced with fluorescently labeled *E. coli* bioparticles (Molecular Probes; Eugene, OR) for an additional 2 h. Subsequently, the bioparticles were aspirated and excess fluorescence was quenched with trypan blue. The remaining fluorescence of the cells was measured on a SpectraMax Gemini EM plate reader (Molecular Devices; Sunnyvale, CA), at 480 nm/520 nm.

### Cellular migration

Cellular migration of macrophages was assessed using a Cytoselect Cell Invasion Assay (Cell Bio Labs, Inc.; San Diego, CA), consisting of a 5-um membrane insert. One million macrophages in serum-free media were added to the membrane insert. The lower well of the migration plate contained FBS supplemented cell culture media. The cell suspension was incubated for 24 h at 37 **°**C. Following incubation, the cells that had migrated into the lower well were lysed, using a fluorescent lysis buffer. Fluorescence of the macrophages was measured on a SpectraMax Gemini EM plate reader (Molecular Devices; Sunnyvale, CA), at 480 nm/520 nm.

### Cytokines analysis

Cytokine levels from macrophage cell lysates were assessed using a mouse cytokine array kit (R&D Systems; Minneapolis, MN). The macrophages were incubated with either control media, 50 ng/ml of lipopolysaccharide (LPS) (Sigma Aldrich; St. Louis, MO), or 100 uM paraquat dichloride hydrate (Sigma Aldrich; St. Louis, MO) for 24-h. Dosages were selected based on previous reports [42, 43]. Following incubation, the cells were washed with PBS and lysed with the assay lysis buffer. The lysates were then mixed with a cocktail of biotinylated antibodies and incubated on a nitrocellulose membrane containing 40 different cytokine antibodies. Next, the membranes were washed and incubated with streptavidin-HRP for 30 min and then developed following the manufacturer’s instructions using GE Amersham Imager 600 (GE; Buckinghamshire, UK). The blots were analyzed using ImageJ software (NIH; Bethesda, MD).

### ^59^Fe loading and release

To determine iron release, macrophages were loaded with ^59^Fe and the amount of iron released was monitored by sampling the media over 24-h. One million macrophages were plated in a 6-well tissue plate and incubated with 2 μCi/well of ^59^Fe-NTA complex overnight. The ^59^Fe-NTA complex was generated, as previously described [44]. After iron loading, the cells were washed twice with DPBS to remove iron from the culture media. The media was then replaced with fresh media containing either control, control/500 nM hepcidin, 50 mg/ml apo-transferrin(apo-Tf), apo-Tf/hepcidin, 20 μM DFO, or DFO/hepcidin media. Aliquots of the media (100 μl) were collected at 0, 4, 8, 12, and 24-h. The amount of ^59^Fe within the collected samples was measured on a Beckman Gamma 4000 (Beckman Coulter; Brea, CA).

### Statistical analysis

Macrophage cultures were obtained from cells isolated from three different animals. One set of macrophage cultures was established for each animal; each set consisting of triplicate plating of macrophages. Each experiment was repeated three times on each set of cultures. Statistical analyses were performed using the GraphPad Prism (La Jolla, CA). The results are presented as mean ± SEM. Statistical comparisons were made using an ungrouped *t* test or a one-way analysis of variance (ANOVA) as appropriate. A *p* value of ≤ 0.05 was considered statistically significant.

## Results

### Increased cell proliferation of wildtype macrophages with iron exposure

**Fig. 1:**
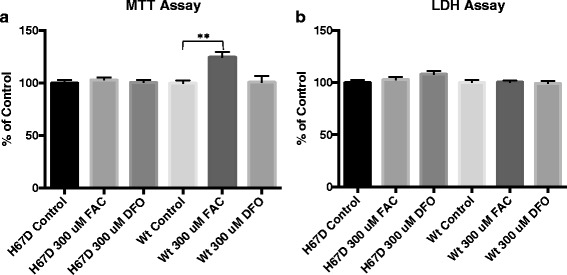
Cell proliferation (MTT) and cytotoxicity (LDH) of macrophages following iron loading and iron chelation. MTT (**a**) and LDH (**b**) assays were carried out to determine cell proliferation and cellular toxicity, respectively. The cells were exposed to control, 300 uM FAC, or 300 μM DFO supplemented media for 24 h. Subsequently, the media was replaced with control media for an additional 24 h, and cell proliferation/cytotoxicity was assessed. Data represent the mean ± SEM from three independent experiments performed with triplicate samples and are compared to all treatment groups for statistical significance using one-way ANOVA. **Compared to control *p* < 0.01

Wildtype macrophages treated with 300 μM FAC had a 24% increase (*p* < 0.0001) in cellular proliferation compared to the non-iron-treated WT group. The addition of iron had no effect on the proliferation of the H67D HFE macrophages. Treatment with the iron chelator DFO had no effect on cell viability of either genotype (Fig. 1a). The manipulation of iron content had no effect on LDH release for either group (Fig. 1b).

### Iron loading induces greater L-ferritin expression in H67D HFE macrophages

**Fig. 2:**
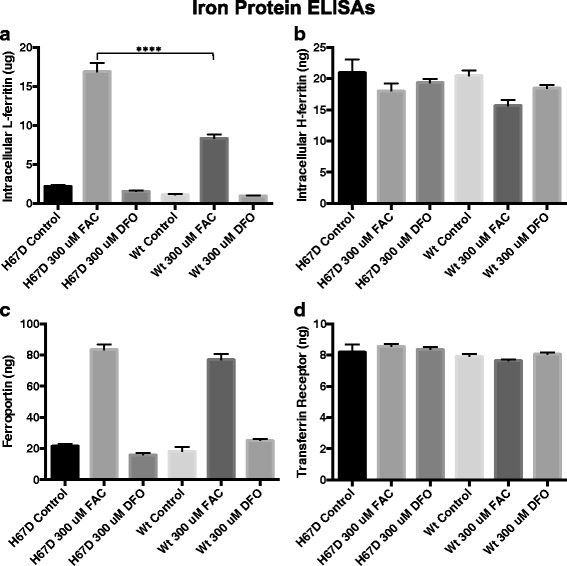
Iron handling protein concentrations in cellular lysates. Macrophages were plated in 100-mm^2^ tissue culture dishes (8 × 10^6^ cells/dish). Following differentiation, the cells were incubated with control, 300 μM FAC, or 300 uM DFO supplemented media for 24 h. Subsequently, the media was replaced with control media for an additional 24 h and then harvested for analysis using an L-ferritin (**a**), H-ferritin (**b**), ferroportin (**c**), or transferrin-receptor (**d**) ELISA. The absorbance of the cell lysates was measured on a SpectraMax 340PC plate reader. Data represent the mean ± SEM from three independent experiments performed each time with triplicate samples and are compared to all treatment groups for statistical significance using one-way ANOVA. ***p < 0.0001

To interrogate intracellular iron handling, we assessed the expression of several key iron regulatory proteins following exposure to iron or an iron chelator. Specifically, we assessed the expression of L-ferritin, H-ferritin, transferrin receptor, and ferroportin in the macrophage lysates. Macrophages were incubated with control, 300 μM FAC or 300 μM DFO media. L-ferritin expression was significantly (*p* < 0.0001) increased following exposure to iron in both WT and H67D macrophages; however, there was 2× the amount of L-ferritin in the H67D cells compared to WT (Fig. 2a; *p* < 0.0001) following iron loading. DFO exposure had no effect on L-ferritin expression in macrophages for either genotype (Fig. 2a). The H-ferritin subunit expression was not affected in either genotype by any of the iron manipulations (Fig. 2b). Ferroportin was detected in untreated control macrophages at the same level across genotypes. In response to iron treatment, both WT and H67D macrophages had similar increases in expression of ferroportin. Exposure to the iron chelator, DFO, had no effect on expression of ferroportin (Fig. 2c). Lastly, high levels of the transferrin receptor were detected in both genotypes and these levels were unchanged by iron loading or iron chelation (Fig. 2d).

### H67D HFE is associated with increased BMP6 secretion

**Fig. 3:**
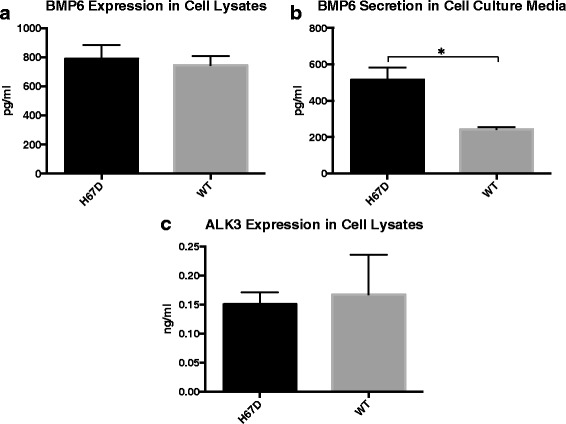
Genotype Effects on BMP6 and its ALK3. Macrophages were plated in 100-mm^2^ tissue culture dishes (8 × 10^6^ cells/dish). Following differentiation, the cell culture media was replaced with fresh control media for 24 h. After 24 h, the media was collected and cells were harvested and lysed for analysis by ELISA. BMP6 expression was measured in the cell lysates (**a**) and secretion in the cell culture media (**b**). ALK3 expression was determined in the cell lysates (**c**). Absorbance was measured on a SpectraMax 340PC plate reader. Data represent the mean ± SEM from three independent experiments performed each time in triplicate. Statistical significance was measured using an unpaired *t* test with Welch’s correction. *p < 0.05

The expression of BMP6 and its receptor ALK3 were assessed in H67D HFE and WT macrophages. BMP6 was measured in both the cell culture media and cell lysates. There was no genotype difference in intracellular BMP6 (Fig. 3a). However, H67D HFE macrophages secreted 112% more (*p* < 0.05) BMP6 compared to WT (Fig. 3b). There was no genotype difference between wildtype and H67D HFE macrophages in the expression of the BMP6 receptor ALK3 (Fig. 3c).

### Iron release from macrophages is mediated by apo-transferrin and DFO

**Fig. 4:**
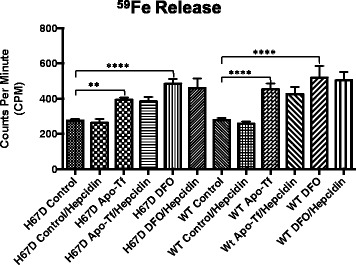
Amount of ^59^Fe released from macrophages following 24 h of ^59^Fe Loading. 2 uCi/well of ^59^Fe was loaded to 1 × 10^6^ macrophages, plated in 6-well tissue culture dishes, overnight. Following overnight incubation, the cells were washed and replaced with normal culture media, or media containing hepcidin, apo-Tf, DFO, apo-Tf and hepcidin, or DFO and hepcidin. 100 μl aliquots were taken at multiple time points but only the 24-h time is shown here. Data represent the mean ± SEM from three independent experiments performed each time in triplicate and are compared to control for statistical significance using two-way ANOVA. **p < 0.01; ****p < 0.0001

Iron release was measured by loading the macrophages with radioactive iron, ^59^Fe, overnight. While no differences in genotype were observed in iron release in the control untreated groups, strikingly, both apo-transferrin and DFO promoted a release of iron previously not observed in macrophages, and in contrast to other cells, this release was not inhibited by hepcidin. Significant changes between the treatment groups were observed, beginning at hour 4 (data not shown). The most significant differences occurred at 24 h (Fig. 4).

### H67D regulates migration, phagocytosis, and cytokine expression in macrophages

**Fig. 5:**
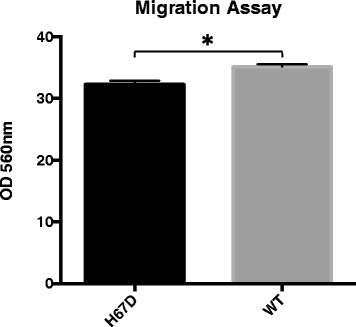
Genotype Affects Cellular Migration. One million macrophages suspended in serum-free media were placed in a 5-μm membrane separated from the chemoattractant, FBS, located in the bottom well. The cells that migrated across the membrane were lysed with a fluorescence lysis buffer. Fluorescence was quantified on a fluorescent plate reader at excitation/emission wavelengths of 490/520 nm. Data represent the mean ± SEM from three independent experiments, each performed in triplicate. Statistical significance was measured using an unpaired t-test with Welch’s correction. *p < 0.05

**Fig. 6:**
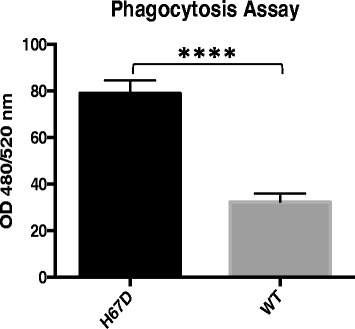
Enhance Phagocytosis Activity by H67D HFE Macrophages. 1 × 10^4^ macrophages were plated in a 96-well plate. 24 h following plating, the cells were then incubated with a fluorescently labeled *E. coli* bioparticle. The fluorescence of the engulfed bioparticles was quantified on a fluorescent plate reader at excitation/emission wavelengths of 480/520 nm. Data represent the mean ± SEM from three independent experiments, performed in triplicate. Statistical significance was measured using an unpaired *t* test with Welch’s correction. *p < 0.0001

To characterize the functional consequences of H67D HFE mutation in macrophages, we assessed several major functions of macrophages; specifically, migration, phagocytosis, and expression of cytokines. To determine whether the H67D HFE genotype affects cellular migration, a chemotaxis assay was performed with FBS as the chemoattractant. Significantly more WT macrophages migrated than H67D HFE macrophages (Fig. 5). The H67D HFE macrophages also had approximately twice the amount of phagocytic activity (*p* < 0.0001) compared to the wildtype macrophages (Fig. 6).

To determine whether H67D HFE alters cytokine expression, 40 different cytokines were analyzed using a cytokine array panel on macrophages under 3 different conditions; control, 50 ng/ml LPS or 100 μM paraquat. Of the 40 cytokines analyzed, 15 were detected within the macrophage cell lysates and six (M-CSF, TREM-1, SICAM-1, JE, IL1ra, and MIP2) showed significant differences between genotypes. M-CSF was the only cytokine that showed a significant genotype difference in the cells incubated with control media only (Table 1). The remaining five cytokines had significant genotype differences following exposure of the macrophages to LPS. Cytokines, TREM-1 and SICAM-1, were significantly increased within wildtype macrophages; whereas JE, IL-1ra, and MIP-2 were significantly increased in H67D HFE macrophages (Table 1). Exposure of macrophages to paraquat did not induce any significant differences between the genotypes (data not shown).

**Table 1:** Cytokine expression panel comparing H67D HFE and WT macrophages

Cytokines	Control media treatment	LPS media treatment
	*H67D* vs. *WT*	*H67D* vs. *WT*
sICAM-1	0.97-Fold	0.76-Fold***
IL-1α	0.89-Fold	0.66-Fold
IL-1β	1.21-Fold	0.97-Fold
IL-1ra	1.26-Fold	1.27-Fold*
IL-10	1.82-Fold	1.09-Fold
IL-16	1.18-Fold	0.77-Fold
IL-17	1.55-Fold	1.44-Fold
IL-23	nd	0.48-Fold
KC	0.76-Fold	1.65-Fold
M-CSF	0.77-Fold**	0.90-Fold
JE	1.01-Fold	1.51-Fold****
MIP-2	0.38-Fold	1.76-Fold****
RANTES	1.35-Fold	0.95-Fold
TNF-α	1.41-Fold	1.2-Fold
TREM-1	0.95-Fold	0.6-Fold*

Cell lysates from macrophages conditioned with control media or 50 ng/ml LPS were analyzed on a nitrocellulose membrane containing 40 different cytokines. The membranes were exposed using GE Amersham Imager 600. Of the 40 cytokines, 15 cytokines (represented in the table) had measurable levels of expression in at least one condition. Five of the 15 had genotype-specific responses to LPS and only one had differences in the non-LPS stimulated cells. Data are reported as fold change of H67D macrophages compared to WT. Statistical significance was measured using a one-way ANOVA. *p < 0.05, **p < 0.01, ***p < 0.001. ****p < 0.0001

## Discussion

This study was undertaken because of the role macrophages and microglia play in the engagement of inflammatory responses that impact repair of tissue damage and outcome of disease [17–20]. The H63D variant of the HFE gene is reportedly a disease modifier for a number of neurodegenerative diseases and possibly cancer [7, 45]. In this study, we demonstrate that the H67D HFE genotype impacts a number of critical macrophage functions, such as cytokine profiles, migration and phagocytosis. There were minimal differences in iron handling between the genotypes; however, the significantly greater increase in L-ferritin in the H67D HFE cells following iron exposure indicates a fundamental difference in storing of iron between the genotypes. The differences in BMP6 secretion between the two genotypes could impact iron handling in the body. The HFE protein reportedly upregulates hepcidin levels through the BMP6 pathway [46]. Hepcidin is a major iron regulatory protein and an increase in hepcidin production could result in decreased iron uptake from the gut [47]. The expectation would be that increased hepcidin would decrease the release of iron from the macrophages [48] but in this study, hepcidin did not block the release of iron that was induced by apo-Tf or exposure to iron chelation. The release of iron from macrophages upon exposure to apo-Tf (iron-poor transferrin) can increase the release of iron from macrophages is a significant and novel finding.

HFE genotype impacted fundamental functions of macrophages such as proliferation and survival. WT macrophages, but not H67D HFE macrophages, treated with iron had a significant increase in cellular proliferation. Iron is critical for cell proliferation, including macrophages [49, 50]. The mechanism underlying the differences in proliferation response in the absence of other stimulatory factors is not known but indicates fundamental differences in iron handling. In support of this idea, macrophages of both genotypes loaded iron as indicated by a similar percentage increase in L-ferritin, but the final concentration in L-ferritin was twice the amount in the H67D HFE macrophages compared to wildtype suggesting more iron was stored rather than made bioavailable. An increase in L-ferritin-positive microglia was also reported in the brain in mice carrying the H67D HFE genotype [13]. The iron export protein ferroportin [49, 50] also increased with iron loading as expected [51]: however, the increase was not genotype specific. These data suggest that regulation of iron export was similar between the two genotypes, a notion supported by the iron release experiments. There were no differences between genotype or treatment groups in the levels of iron import protein, transferrin receptor (TfR) (Fig. 2d). The regulation of ferroportin, ferritin, and transferrin receptor are mediated through iron regulatory element/iron regulatory protein system (IRE/IRP) [52, 53]. This post-transcriptional regulation typically results in complimentary expression of proteins, in response to iron changes. Thus, the lack of a predictable response in IRE/IRP-regulated protein expression in macrophages is an area for additional investigation.

A significant function of macrophages is recycling of iron [54]. The secretion of iron from macrophages is mediated by both ferroportin [55] and H-ferritin [56]. However, the regulation of iron secretion is unclear. We demonstrated that iron release is signaled by the presence of apo-Tf and DFO. This finding suggests that circulating transferrin in the serum, which is 70% unsaturated [57], can serve as a mechanism to induce the release of iron from macrophages. We previously reported that apo-Tf can induce iron release from endothelial cells of the blood-brain-barrier (BBB); therefore, the function of apo-Tf to remove iron from cells may be a significant and unrecognized function of this protein [58]. Moreover, the response by the macrophages to release iron when exposed to DFO suggests treatment with iron chelators in clinical settings may also remove iron from macrophages. We attempted to block the release of iron by using hepcidin. Hepcidin binds to ferroportin and limits iron release in a number of cells [59]. The presence of hepcidin in the media did not limit the iron release by DFO or apo-Tf, consistent with other reports of an export system independent of the hepcidin and ferroportin system [60].

Hepcidin secretion is regulated by stimulation of BMP6 through its receptor ALK3 [46]. The cellular levels of BMP6 and ALK3 were not affected by genotype but secreted BMP6 was elevated in the H67D HFE cells. The combination of normal levels in the cell plus increased expression of BMP6 suggests an increased synthesis of this protein in the H67D cells. Higher levels of secreted BMP6 could result in more stimulation of ALK3 receptors on hepatocytes and translate to higher levels of circulating hepcidin. However, increased circulating hepcidin may not limit the iron release from macrophages as indicated in our study and others [60]. In contrast, however, hepcidin production is reduced by IL-1 receptor antagonist (IL-1ra), which was elevated in the H67D HFE macrophages compared to WT. These results indicate a complex signaling system via cytokines for hepcidin production that is genotype dependent.

By secreting cytokines, macrophages play a key role as inflammatory cells and are critical in the innate immune response. HFE knock out mice have an attenuated immune response [61]. The decrease in expression of cytokines, TREM-1 and sICAM1 by the H67D HFE macrophages would be consistent with a decreased inflammatory response and can lead to disease promotion. For example, decreased expression of TREM-1 in microglia has been correlated with decreased clearance of Aβ plaques in models of Alzheimer’s disease [62]. Furthermore, increased expression of SICAM-1 has been found to decrease the formation of Aβ plaques through microglia secretion of neprilysin, the Aβ-degrading enzyme [63]. These studies further support the contribution of H67D HFE mutation in the progression of neurodegenerative disease.

As part of the immune response, macrophages migrate to different infection sites and engage in phagocytosis. The H67D HFE macrophages have increased phagocytic ability compared to WT (Fig. 6) but slower migration rates than wildtype macrophages (Fig. 5). We previously reported that increasing iron content in a rat microglial cell line was associated with a decrease in phagocytic activity following LPS stimulation [24]. Given the number of different conditions in our previous study, direct comparisons to the current study are difficult, but clearly, iron status impacts phagocytosis. Furthermore, two cytokines involved in chemotaxis, macrophage inflammatory protein 2 (MIP-2) and JE, were elevated in the H67D HFE macrophages. We also previously reported that monocyte chemoattractant protein-1 (MCP-1) is elevated in amyotrophic lateral sclerosis patients with H63D HFE [64], as well as in SH-SY5Y neuroblastoma cells transfected with the H63D HFE mutation [65]. Although we measured expression and not secretion, intracellular expression of cytokines has been found to correlate to cytokine secretion [66, 67]. These findings support the concept that the HFE mutation may promote increased migration of lymphocytes and microglia through the secretion of chemoattractant proteins.

## Conclusions

In general, these results indicate that the H67D HFE genotype impacts macrophage phenotype. These studies identify areas for future studies into HFE impact via macrophage and microglia dysfunction, in the context of neurodegenerative disorders, such as Alzheimer’s, ALS, and Parkinson’s as well as many types of cancer.

## Abbreviations

ALK3/BMPI: Bone morphogenetic protein type I receptor
ALS: Amyotrophic lateral sclerosis
ANOVA: Analysis of variance
Apo-Tf: Apo-Transferrin (iron poor transferrin)
BBB: Blood-brain-barrier
BMM: Bone marrow macrophage
BMP: Bone morphogenetic protein
DFO: Deferoxamine
DMEM: Dulbecco’s Modified Eagle’s Medium
DPBS: Dulbecco’s phosphate-buffered saline
ELISA: Enzyme-linked immunosorbent assay
FAC: Ferric ammonium citrate
FBS: Fetal bovine serum
Il-1ra: Interleukin 1 receptor antagonist
IRE: Iron regulatory element
IRP: Iron regulatory protein
LDH: Lactate dehydrogenase
LPS: Lipopolysaccharide
MCP-1: Monocyte chemoattractant protein-1
M-CSF: Macrophage-colony stimulating factor
MIP-2: Macrophage inflammatory protein-2
MTT: 3-(4,5-Dimethylthiazol-2-Yl)-2,5-diphenyltetrazolium bromide
SEM: Standard error of mean
WT: Wildtype
